# Supplementary material for: Rabies as a Public Health Concern in India—A Historical Perspective
Source: Trop Med Infect Dis. 2020 Oct 21;5(4):162. doi: 10.3390/tropicalmed5040162 (PMC7709690; doi:10.3390/tropicalmed5040162)
Supplement: Supplementary file 1 [file tropicalmed-05-00162-s001.pdf]

Supplementary

Rabies as a public health concern in India – a historical perspective

1. Compilation of historical animal rabies incidence in British India

Owing to how disease statistics were presented in annual administration reports of the Civil Veterinary Department in British India, it is not straightforward to collate animal rabies statistics. The reports include disease statistics in three possible forms – the most accessible form was a table of summary statistics (Table II) of all major infectious diseases of animals appearing at the end of annual reports; there were also descriptive summaries of individual diseases in the main text; and summaries of cases presented to or diagnosed at regional veterinary colleges, where these existed. In later years, there are also reports of number of brain samples tested and confirmed rabies positive at regional diagnostic laboratories.

Statistics from all sections often do not match or are difficult to disentangle. Table II did not include rabies cases from veterinary colleges – for instance in Madras Presidency, the first case of rabies (one dog from Madras city) is reported in Table II only in 1922-23 [1], although rabies was diagnosed at the Madras Veterinary College over a decade earlier (1911-12) [2]. Later reports further complicate matters by splitting cases into those that were confirmed by laboratory diagnosis (detection of Negri bodies in brain samples), those for which laboratory confirmation was not sought due to characteristic rabies symptoms, as well as the number of animals which died during or after a course of post-exposure prophylaxis [3].

In addition, reporting of animal rabies cases was not mandatory [4]. Consequently, many provinces did not collect rabies statistics systematically or explicitly include case numbers, despite an acknowledgement that it was a problem within their administrative boundaries. In many instances, rabies cases are combined with cases of other less common animal diseases under the broad heading of 'Other' diseases. Finally, entire annual reports or statistical tables from those reports were not available for some years on the Medical History of British India website.

Table S1 presents the annual number of rabies cases diagnosed in all domestic and wildlife species - dogs, cats, cattle, buffalo, goats, sheep, camels, horses, donkeys, mules, pigs, elephants, mongoose, jackals and foxes. These numbers include cases reported in Table II of CVD annual reports, additional cases mentioned in the main text of reports that have not been included in Table II, number of confirmed cases (by diagnostic tests or based on symptoms) reported from veterinary colleges / schools and other districts that have not been included in Table II, and the number of samples reported to have tested positive from diagnostic laboratories. While every attempt has been made to avoid double-counting of cases and to include all reported cases, it is possible that the true rabies incidence is slightly different from those presented in Table S1 (and consequently, Figures 1 and 2).

British provinces where veterinary colleges / schools functioned are:

1. Bombay province – Bombay Veterinary College
2. Punjab province – Lahore Veterinary College
3. Madras presidency – Madras Veterinary College
4. Bengal province – Bengal Veterinary College, Calcutta
5. Bihar province – Bihar Veterinary College, Patna
6. Burma province – Insein Veterinary School, Rangoon

References

1. (360) Page 12. In: Annual Administration report of the Civil Veterinary Department, Madras Presidency for 1922-23 [Internet]. 1923 [cited 21 Jul 2020] p. 22. Available: https://digital.nls.uk/indiapapers/browse/archive/75699817
2. (48) Page 19. In: Report on the operations of the Civil Veterinary Department, Madras. Recording, with remarks, the - during 1911-12 [Internet]. 1912 [cited 21 Jul 2020] p. 23. Available: https://digital.nls.uk/indiapapers/browse/archive/75698863
3. (371) Page 63. In: Annual Administration Report of the Madras Civil Veterinary Department for the year 1928-29 [Internet]. 1929 [cited 21 Jul 2020] p. 98. Available: https://digital.nls.uk/indiapapers/browse/archive/75718016
4. (53) Page 7. In: Annual report of the Civil Veterinary Department Bihar and Orissa for the year 1923-24 [Internet]. 1924 [cited 23 Jul 2020] p. 17. Available: https://digital.nls.uk/indiapapers/browse/archive/75900869

Table S1. Annual rabies incidence in all animal species between 1887-1888 and 1950-51 (denoted 1888 and 1951, respectively) in all British provinces in India and the princely state of Manipur. Statistics were compiled from annual reports of the Civil Veterinary Department of the colonial British Government of India, available at https://digital.nls.uk/indiapapers/.

Year	UP	P	Sind	R	Be	Bo	M	CP	Bu	Bal	NWFP	EBA*	MS	BO**
1888	NA	NA	NA	NA	NA	NA	NA	NA	NA	NA	NA	NA	NA	NA
1889	NA	NA	NA	NA	NA	2	NA	NA	NA	NA	NA	NA	NA	NA
1890	NA	NA	NA	NA	NA	8	NA	NA	NA	NA	NA	NA	NA	NA
1891	NA	NA	NA	NA	NA	2	NA	NA	NA	NA	NA	NA	NA	NA
1892	NA	18	NA	NA	NA	11	NA	NA	NA	NA	NA	NA	NA	NA
1893	NA	10	NA	NA	NA	9	NA	NA	NA	NA	NA	NA	NA	NA
1894	NA	14	NA	NA	NA	20	NA	NA	NA	NA	NA	NA	NA	NA
1895	NA	13	NA	NA	NA	13	NA	NA	NA	NA	NA	NA	NA	NA
1896	NA	9	NA	NA	3	15	NA	NA	NA	NA	NA	NA	NA	NA
1897	NA	29	NA	NA	3	15	NA	NA	NA	NA	NA	NA	NA	NA
1898	NA	40	NA	NA	3	21	NA	NA	NA	NA	NA	NA	NA	NA
1899	NA	42	NA	NA	4	87	NA	NA	NA	NA	NA	NA	NA	NA
1900	NA	NA	NA	NA	5	32	NA	NA	NA	NA	NA	NA	NA	NA
1901	NA	NA	NA	NA	18	NA	NA	NA	NA	NA	NA	NA	NA	NA
1902	NA	NA	NA	NA	6	NA	NA	NA	NA	NA	NA	NA	NA	NA
1903	NA	NA	15	NA	20	7	NA	NA	NA	NA	NA	NA	NA	NA
1904	0	0	9	0	25	10	NA	NA	NA	NA	NA	NA	NA	NA
1905	0	4	24	0	36	NA	NA	2	NA	NA	NA	NA	NA	NA
1906	30	17	4	7	11	NA	NA	NA	NA	NA	NA	NA	NA	NA
1907	12	10	4	6	55	1	NA	3	NA	NA	NA	NA	NA	NA
1908	6	26	10	4	75	NA	12	NA	NA	NA	NA	NA	NA	NA
1909	22	0	54	4	25	77	15	NA	NA	NA	NA	NA	NA	NA
1910	2	0	21	6	48	86	NA	NA	NA	NA	NA	0	NA	NA
1911	0	0	19	3	79	71	0	NA	NA	NA	NA	224	NA	NA
1912	41	0	11	9	81	50	1	NA	NA	1	NA	42	NA	NA
1913	149	0	22	2	60	75	1	NA	NA	NA	NA	25	NA	NA
1914	85	0	43	5	95	72	12	NA	NA	NA	NA	39	NA	NA
1915	137	0	50	21	200	74	0	NA	NA	NA	NA	144	3	NA
1916	175	0	51	14	107	59	37	NA	8	NA	NA	44	9	NA
1917	68	0	NA	4	62	NA	22	NA	11	NA	NA	49	7	NA
1918	98	0	48	0	74	73	70	NA	6	1	NA	302	7	NA
1919	104	0	52	5	121	54	143	NA	13	NA	NA	42	0	NA
1920	138	6	33	8	96	76	105	NA	2	NA	NA	94	NA	33

1921	102	0	13	3	91	48	113	NA	9	NA	NA	192	4	49
1922	126	0	15	1	NA	58	104	NA	NA	NA	NA	199	5	NA
1923	132	0	37	1	NA	65	103	NA	4	NA	NA	171	11	45
1924	134	2	42	4	182	90	111	NA	0	NA	NA	232	13	72
1925	193	0	28	16	NA	67	97	NA	3	NA	NA	232	16	62
1926	174	0	48	21	94	49	97	33	1	NA	NA	234	24	95
1927	170	0	32	5	100	30	108	25	4	NA	NA	297	29	108
1928	162	0	47	15	109	25	78	NA	3	NA	NA	376	13	64
1929	105	0	41	1	NA	28	109	NA	2	NA	40	232	0	79
1930	123	0	51	2	92	41	306	NA	NA	NA	103	283	2	68
1931	76	0	61	5	61	25	187	NA	NA	NA	60	200	NA	75
1932	138	0	36	10	60	75	138	NA	1	NA	45	210	NA	43
1933	159	NA	75	11	115	73	175	3	16	NA	80	398	NA	63
1934	196	NA	67	14	147	37	NA	NA	20	NA	82	138	NA	84
1935	143	NA	62	23	88	102	NA	NA	36	NA	95	NA	NA	59
1936	231	NA	85	29	950	69	NA	NA	22	NA	48	227	NA	65
1937	241	NA	32	32	123	88	NA	NA	11	NA	29	260	NA	65
1938	234	NA	50	8	NA	73	NA	9	12	NA	57	202	NA	62
1939	259	NA	56	58	241	54	NA	36	11	NA	98	150	NA	73
1940	277	NA	39	37	NA	54	NA	42	16	NA	67	223	NA	81
1941	241	NA	60	NA	NA	71	NA	42	11	NA	44	249	NA	41
1942	284	NA	53	NA	NA	NA	NA	NA	NA	NA	NA	NA	NA	56
1943	NA	NA	66	NA	NA	NA	NA	NA	NA	NA	NA	NA	NA	22
1944	NA	NA	55	NA	NA	NA	NA	NA	NA	NA	49	NA	NA	35
1945	NA	NA	41	NA	NA	NA	NA	NA	NA	NA	37	92	NA	43
1946	NA	NA	48	NA	68	NA	NA	NA	NA	NA	9	113	NA	27
1947	NA	NA	NA	NA	60	NA	NA	NA	NA	NA	NA	165	NA	NA
1948	NA	NA	NA	NA	56	NA	NA	NA	NA	NA	NA	180	NA	34
1949	NA	NA	NA	NA	69	NA	NA	NA	NA	NA	NA	158	NA	12
1950	NA	NA	NA	NA	111	NA	NA	NA	NA	NA	NA	109	NA	25
1951	NA	NA	NA	NA	90	NA	NA	NA	NA	NA	NA	NA	NA	NA

British Provinces: UP – United Provinces, P – Punjab, R – Rajputana, Be – Bengal, Bo – Bombay, M – Madras, CP – Central Provinces, Bu – Burma, Bal – Baluchistan, NWFP – North-West Frontier Province, EBA – Eastern Bengal and Assam, BO – Bihar and Orissa; Princely states: MS – Manipur state; *1907-08 to 1910-11 – Eastern Bengal and Assam provinces; 1912-13 to 1949-50 – Assam only; **1911-12 to 1935-36 – Bihar and Orissa provinces; 1936-37 to 1949-50 – Bihar only

Publisher's Note: MDPI stays neutral with regard to jurisdictional claims in published maps and institutional affiliations.

© 2020 by the authors. Submitted for possible open access publication under the terms and conditions of the Creative Commons Attribution (CC BY) license (http://creativecommons.org/licenses/by/4.0/).
